# Erythrocyte transketolase activity measured on the Gemstar discrete analyser

**DOI:** 10.1155/S1463924684000316

**Published:** 1984

**Authors:** J. E. Buttery, B. R. Chamberlain, C. R. Milner

**Affiliations:** Department of Clinical Chemistry The Queen Elizabeth Hospital Woodville South Australia 5011 Australia


Journal of Automatic Chemistry, Volume 6, Number 3 (July–September 1984), pages 161–162

### Erythrocyte transketolase activity measured on the Gemstar discrete analyser

**J. E. Buttery, B. R. Chamberlain and C. R. Milner**

*Department of Clinical Chemistry, The Queen Elizabeth Hospital, Woodville, South Australia 5011, Australia*

### Introduction

The NADH-dependent method of Smeets *et al.* [1] for erythrocyte transketolase (TK, EC 2.2.1.1) is a kinetic assay which measures the rate of decreasing absorbance of NADH at 340 nm. The enzyme activity can be enhanced *in vitro* by the addition of thiamine pyrophosphate (TPP), and this increase, expressed as a percentage, is called the TPP effect.

The TK assay has been automated using a centrifugal analyser [2] and the LKB Reaction Rate Analyser [3], but both techniques require deproteinization. A direct adaptation of Smeets's procedure to the Gilford System 5, a discrete analyser, has also been published [4]. Although the latter method is simple and economical, it is time-consuming for large-batch analyses, as the System 5 measures one test at a time.

Recently, a discrete 12-sample analyser, the Gemstar (Electro-Nucleonics, Fairfield, New Jersey 07006, USA) was introduced. The procedure of Smeets *et al.* was adapted for this machine and this paper describes the details and compares the results obtained with those derived by the manual method of Smeets on the Pye Unicam SP8000 spectrophotometer.

### Materials and methods

All reagents are similar to those of Smeets *et al.*, with modifications as previously described [4]. The reagents are mixed prior to the assay and the proportions sufficient for five specimens, each assayed for TK and its TPP effect are as follows:

| | |
|---|---|
| Ribose-5-phosphate, 37·5 mmol/l in tris buffer | 3 ml |
| Tris buffer, 0·1 mol/l, pH 7·8 at RT | 3 ml |
| NADH solution, 0·01 mol/l in tris buffer | 150 μl |
| Glycerol-3-phosphate/triosephosphate isomerase (GDH/TIM), 2 mg/ml Boehringer | 30 μl |

The reagent mixture is divided into 2 × 2·8 ml portions. To one portion is added 1·4 ml tris buffer for the TK assay (Reagent A). To the other is added 1·2 ml tris buffer and 0·2 ml TPP solution (0·01 mol/l thiamine pyrophosphate in tris buffer) for the activated TK assay (Reagent B).

### *Sample preparation*

Blood is collected in a heparinized tube and processed as described by Smeets *et al.* The haemoglobin concentration is determined by the cyanomethaemoglobin method and the final concentration is then adjusted to 30 g/l with saline and stored at −20°C. Just prior to assay, the specimens are thawed at room temperature, mixed and centrifuged to obtain haemolysate free of stroma.

### *Gemstar*

This is a compact table-top discrete clinical chemistry analyser which can perform end-point and kinetic tests simultaneously on 12 samples. In addition to 21 pre-programmed chemistries, there are nine open channels which allow the operator to programme other tests, for example TK. After presenting the reagent and specimens to the Gemstar, the system takes over completely and provides the results in a print-out form. Flagged messages alert the operator to abnormal occurrences during the run.

### *Reagent and sample loading*

Up to 12 cuvettes may be placed on the Gemstar plastic holder. Tris buffer (700 μl) is added into the first cuvette which acts as the blank. Reagent A is then pipetted into the next five cuvettes, followed by Reagent B into another five cuvettes. The cuvettes are then transferred to the *shuttle assembly* of the Gemstar analyser. The haemolysate specimens are placed in sample cups on the Gemstar pipettor assembly and 10 μl aliquots aspired into the pippetes. The specimens are than simultaneously added into the cuvettes to initiate the reaction.

### *Operation mode*

The Gemstar is set for batch-mode operation and the type of calculation on 'Factor two point' (TC = 7); this is a kinetic two-point assay. The reaction time between the two readings is 15 min, with the first measurement taken after 20 min. The parameters for the TK assay on the Gemstar are:

| Open channel | | |
|---|---|---|
| *Parameter* | *Value* | *Unit* |
| Type of calculation (TC) | 7 | |
| Temperature | 37 | °C |
| Units | 0 | Not specified |
| Decimal places | 3 | |
| Number of readings | 2 | |
| Pre-incubation time 1 | 60 | second(s) |
| Pre-incubation time 2 | 60 | s |
| Direction of reaction | 0 | decreasing |
| Filter wheel position 1 | 1 | 340 nm |
| Filter wheel position 2 | 0 | |
| First read time | 1200 | s |
| Time between readings | 900 | s |
| Maximum reference absorbance | 0 | |
| Sector size | 0 | |

| | |
|---|---|
| Standard error of estimate | 0 |
| Auxilliary limit | 2 |
| Factor | 25·4 |
| Maximum delta absorbance | 0 |
| Minimum reagent absorbance | 0 |
| Maximum printable result | 3 |

The above parameters are retained in the Gemstar memory provided that the instrument is not switched off. The reaction, once initiated, will proceed to completion with the TK and activated TK results printed-out after 35 min.

### Results

### Comparison between two methods

The results of TK and activated TK by Gemstar and the manual method of Smeets *et al.* show that the correlation for the TK assay ($r=0{\cdot}98$) and the least squares regression according to Deming [5] is $y=0{\cdot}85x+0{\cdot}144$. Similarly for the activated TK assay, $r=0{\cdot}98$ and the line of regression is $y=0{\cdot}90x+0{\cdot}048$.

Further analytical data on the results by the two methods are given in table 1.

*Table 1. Analytical data of TK activities determined by the two methods.*

| | TK | | Activated TK | |
|---|---|---|---|---|
| | Gemstar | Manual | Gemstar | Manual |
| Mean (U/g Hb) | 0·84 | 0·82 | 0·93 | 0·97 |
| SD (U/g Hb) | 0·32 | 0·38 | 0·32 | 0·36 |
| Actual range | 0·55–1·95 | 0·41–2·16 | 0·59–2·12 | 0·63–2·31 |
| *N* | 22 | 22 | 22 | 22 |

### Precision

The within-day precision for the Gemstar procedure on two samples is shown in table 2. Owing to specimen instability, the day-to-day precision could not be performed.

*Table 2. Within-day TK precision on the Gemstar.*

| | Within-day precision | |
|---|---|---|
| | Specimen 1 | Specimen 2 |
| Mean (U/g Hb) | 0·531 | 0·811 |
| SD (U/g Hb) | 0·026 | 0·024 |
| CV (%) | 4·9 | 3·0 |
| *N* | 10 | 10 |

### Discussion

To ensure that the measurement of TK activity occurs during the linear part of the reaction, the first absorbance was not measured until 20 min after the addition of haemolysate to the substrate. This time also ensures the complete activation of TK by thiamine [4]. The TK reaction was then monitored for 15 min as according to Smeets *et al.*

The calculation of enzyme activity using the 'Factor Two Point' approach obviated the need to programme certain parameters into the Gemstar. These included the maximum reference absorbance, sector size, standard error of estimate, maximum delta absorbance and minimum reagent absorbance. This is fortunate as the instruction manual does not contain information as to how these parameters are to be used.

The *pre-incubation time 1* was kept to a minimum of 60 s so that samples may be introduced without the need for a long reagent pre-incubation to attain the reaction temperature.

The factor of 25·4 takes into account the 15 min read time and 30 g/l haemoglobin concentration. Results are printed without units as there is no code for U/g Hb on the Gemstar.

Erythrocyte transketolase activity is generally difficult to measure in routine laboratories [3]. The authors consider that Smeets's procedure is probably the most specific and convenient method, as was also noted by Bayoumi and Rosalki [6]. This method has been automated on the Gilford System 5 discrete analyser and is now also successfully adapted to the Gemstar, a relatively new and inexpensive analyser.

The reference range for erythrocyte TK activity on the Gemstar is 0·6 to 1·3 U/g Hb, and the range for the TPP effect is 2–25%.

### Acknowledgements

The authors wish to thank Dr M. L. Wellby for his interest and advice in the preparation of this paper.

### References

1. Smeets, E. H. J., Muller, H. and DeWael, J., *Clinica Chimica Acta*, **33** (1971), 379.
2. Van Zanten, A. P., Beijer, C., Mairuhu, M. W. and Van Den Ende, A., *Clinical Chimica Acta*, **105** (1980), 303.
3. Leunis, J. C., Van Riet, C. and Brauman, J., *Clinical Chemistry*, **28** (1982), 391.
4. Milner, C. R., Buttery, J. E. and Chamberlain, B. R., *Journal of Automatic Chemistry*, **4** (1982), 183.
5. Wakkers, P. J. M., Hellendoorn, H. B. A., Op De Weegh, G. J. and Heerspink, W., *Clinica Chimica Acta*, **64** (1975), 173.
6. Bayoumi, R. A. and Rosalki, S. B. *Clinical Chemistry*, **22** (1976), 327.

### INTERNATIONAL MEETING ON PRODUCT DESIGN ASSURANCE IN ENGINEERING

*11–13 June 1985, Wembley Conference Centre, London*

The Society of Environmental Engineers' 1985 conference at Wembley will cover Specifications, Techniques and Case studies.

In conjunction with the conference there will be a major exhibition of environmental equipment.

*Details from Mrs Helen Gibbons, Society of Environmental Engineers Secretariat, Owles Hall, Buntingford, Hertfordshire, UK. Tel.: 0763 71209.*

